# Improving undergraduate teaching of chronic wounds with three-dimensional silicone models: a Finnish questionnaire-based cohort study

**DOI:** 10.1093/skinhd/vzag115

**Published:** 2026-07-14

**Authors:** Alexander Salava, Johanna Mandelin, Kirsi Isoherranen

**Affiliations:** Department of Dermatology and Allergology, Helsinki University Hospital and University of Helsinki, Helsinki, Finland; Department of Dermatology and Allergology, Helsinki University Hospital and University of Helsinki, Helsinki, Finland; Department of Dermatology and Allergology, Helsinki University Hospital and University of Helsinki, Helsinki, Finland

## Abstract

Three-dimensional silicone wound models may improve teachings of chronic wounds and increase learning outcomes in undergraduate education better preparing students for clinical practice. The main benefits reported were the practice of clinical skills, new perspectives, recognition of different types of wounds, and the possibility of practicing wound palpation, measurement and cavity probing. The wound models seemed to increase students’ learning motivation and were easily integrated into the course curriculum with traditional teachings, e.g. lectures or bedside teaching.

Dear Editor, Dermatology education has seen substantial developments in recent years such as virtual reality and artificial intelligence (AI)-based teachings.^1^ Nevertheless, learning of clinical examination, visual skills and palpation of skin lesions remain core curricular components. Modern moulages, for example three-dimensional (3D) silicone models, have a long tradition in simulation-based training and have been used in dermatology, as well as other medical specialties.^2^

The burden of chronic wounds is increasing worldwide, posing demands to wound-care education amongst medical students.^3^ Possibilities for bedside training are limited, addressing the need for alternative learning methods. Simulation relates especially to the diagnosis and management of chronic wounds which are often considered particularly challenging to teach. For example, discrimination of different types of wounds requires ample repetitions, recognition of wound variations, and also training of tactile skills such as palpation, measuring and probing.

We aimed to investigate the usefulness of 3D silicone wound models integrated into traditional and online undergraduate teachings of dermatology.

Undergraduate medical students attending dermatology courses were given the opportunity to practice clinical examination on 3D silicone wound models (Medical FX^®^, Kirchlinteln, Germany). After traditional lectures, bedside teachings and online preliminary tasks, students were offered voluntary 3D training sessions lasting 45–60 min. In total, 162 students took part in dermatology courses during 2024–25, from which 53 (32.7%) participated in the 3D sessions.

We used seven hand-made 3D silicone wound models representing different wound types (1, black-necrotic wound; 2, inflamed wound; 3, wound with fibrinous exudate; 4, deep wound with cavities; 5, granulating wound; 6, hypergranulating wound; and 7, ­epithelializing wound). During the sessions, students were given various tasks, for example discrimination of surface and structures, border, signs of inflammation, and wound measurement, probing of cavities and wound palpation (Figure 1). In addition, important assignments included recognition of the type of wound, possible aetiology, relevant further investigations and local wound care.

**Figure 1:**
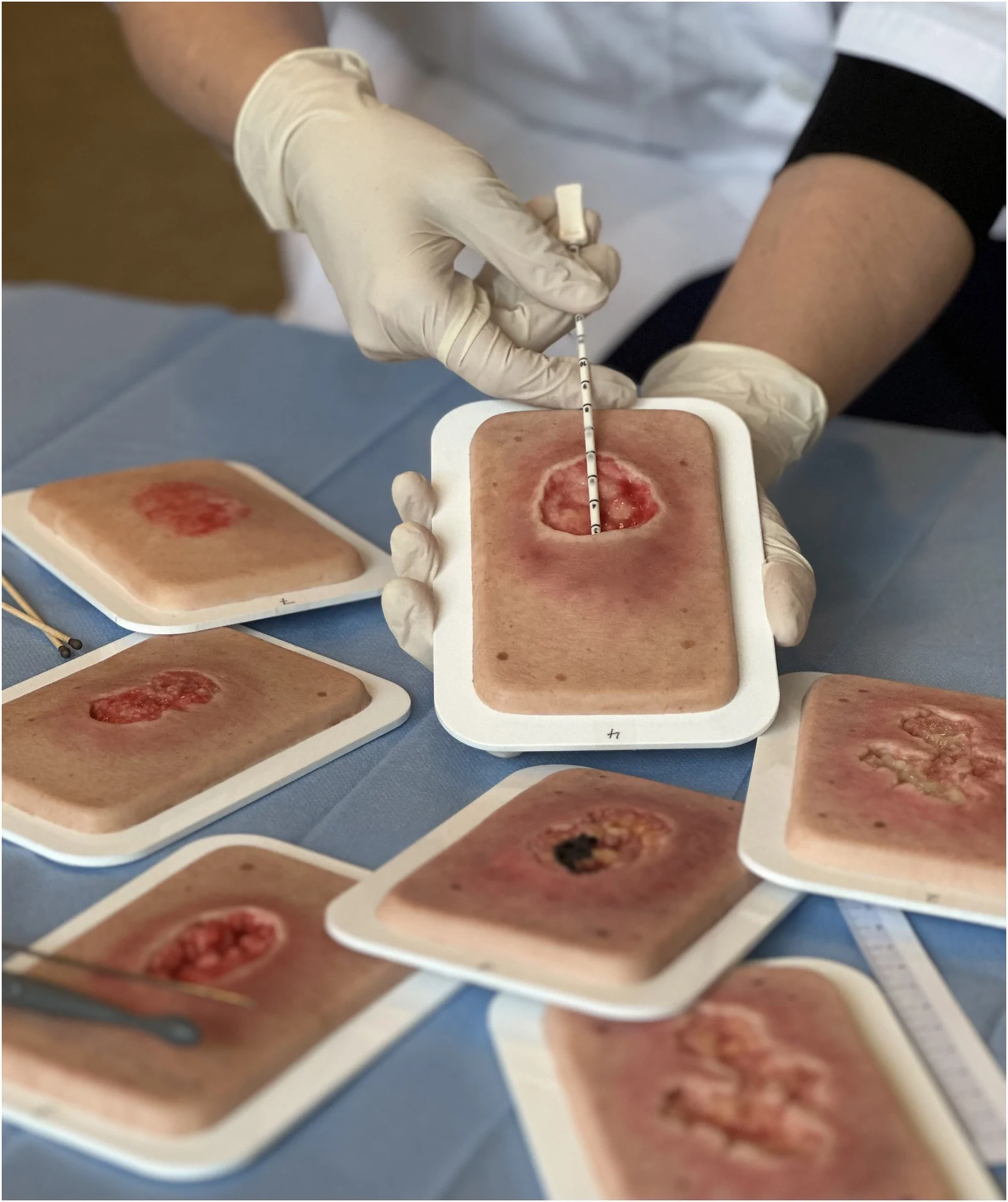
The student is training wound probing on a three-dimensional silicone wound model (plastic probe with measurement scale). On the table are models of different wound types, which can be examined and palpated. There is also a measuring tape to practise measurement and accurate noting of wound extent. Other examples of skills that can be practised: curettage of wound cavities can be practised with a dermal curette (front left), probing of cavities with a metal probe (front left) and cauterization of hypergranulating wounds with silver nitrate sticks (back left).

Students explored a variety of wound types and practised clinical examination with a 3D perspective (e.g. surface topography, wound ground and other key features). After the sessions, students were asked to fill in a questionnaire about the practicability and usefulness of 3D models. The questionnaire included five questions on a 5-point Likert scale to measure students’ opinions regarding functionality, curricular integration and learning effects. In addition, the sessions were scored with a general grade and characterized with two voluntary open questions (Tables 1, 2).

**Table 1:** Questions and results of the quantitative analyses

Question	Results (*n* = 53) [mean, SD (median, IQR, range)]
1. The 3D models increased learning outcomes regarding chronic wounds	4.47, 0.64 (5.00, 4.00–5.00, 2–5)
2. The 3D models provided new perspectives about the appearance of different types of chronic wounds	4.64, 0.52 (5.00, 4.00–5.00, 3–5)
3. The 3D models were useful additions to traditional teachings such as lecture and bedside teaching	4.77, 0.47 (5.00, 5.00–5.00, 3–5)
4. The 3D models improved learning experiences of digital self-study material	4.02, 0.72 (4.00, 4.00–4.50, 2–5)
5. The 3D models had a positive effect on learning motivation	4.23, 0.67 (4.00, 4.00–5.00, 2–5)
6. What general grade would you give the concept of integrating 3D models into dermatology teaching?	8.76, 0.95 (9.00, 8.00–9.00, 4–10)

A total of 53 undergraduate students answered the survey questions. The answer options for questions 1–5 were on a 5-point Likert scale (strongly agree, agree, neutral, disagree, strongly disagree). A general grade for the teaching sessions (question 6) was given on a scale of 10 (best) to 0 (worst). 3D, three-dimensional; IQR, interquartile range.

**Table 2:** Qualitative analysis of open questions included the manual count of key words and concepts (e.g. probing or palpation)

Question	Qualitative analysis [absolute number of answered key words (percentage)]
From a teaching perspective, what were the advantages of using 3D silicone models? How did they enhance your learning experience compared with other methods?	Palpation and probing skills: 34/53 (64%)
	Visual skills (visualization): 31/53 (58%)
	Fun and motivation: 29/53 (55%)
	Clinical skills, 3D perspective: 26/53 (49.1%)
	Possible to see different types of wounds: 19/53 (36%)
	Practice (repetitions): 15/53 (28%)
	Real-life imitation: 11/53 (21%)
	Different types of wounds side-by-side: 9/53 (17%)
What aspects of the 3D silicone models could be improved to enhance the learning experience? How would you suggest making these improvements?	Needs to be used more in teachings, more models: 39/53 (74%)
	Including background information (patient history, wound site): 31/53 (58%)
	Models also from skin tumors: 26/53 (49%)
Artificial intelligence-based (CurreChat^®^)^a^ analysis and summary of both open questions	Advantages of using 3D models:
	Enhanced visual and tactile learning, active learning and interactivity, memory and perception, comprehensive representation
	Areas for improvement:
	Contextual information, variety and expansion, clarity and detail, collaborative learning

In addition, an artificial intelligence-based qualitative analysis was conducted using the university’s own program (CurreChat^®^), which utilizes large language models developed by OpenAI^®^. The first line prompts were the study title and messages were all open questions and students’ answers.

^a^ https://blogs.helsinki.fi/currechat/, https://openai.com. 3D, three-dimensional.

The results were analysed with standard descriptive statistics (SPSS version 29.0.0.0; IBM, Armonk, NY, USA) and presented as the mean (SD) of all answers. A qualitative analysis was carried out, which included the manual counting of key words (e.g. the word ‘probing’). The absolute numbers of words mentioned were ranked and the percentage of students that had used the word ­calculated. In addition, a qualitative analysis was conducted using the university’s own generative AI tool (CurreChat^®^, ­https://blogs.helsinki.fi/currechat/), which utilizes large language models developed by OpenAI^®^. The rationale for using this complementary AI-based analysis was that it would possibly reveal new insights and categorization of the questionnaire data undetectable by manual coding. The first-line prompts (initialization) were the study title, and the messages were all the open questions compared with students’ answers (see Supporting Information).

In general, students were very satisfied with the practicability and functionality of the models and 3D training sessions (Table 1). They believed the models increased learning outcomes [mean (SD) 4.47 (0.64)] and provided new perspectives about the appearance of different wound types [mean (SD) 4.64 (0.52)]. Students also found that the models were useful additions to traditional [mean (SD) 4.77 (0.47)] and online teachings [mean (SD) 4.02 (0.72)]. Most students believed that teachings with 3D models had a positive effect on learning motivation and represented an enjoyable learning experience [mean (SD) 4.23 (0.67)].

The qualitative analysis showed that the main advantages of the models were the development of clinical skills (*n* = 26/53 students; 49%), repeated practice (*n* = 15/53 students; 28%), enhancement of visual skills (*n* = 31/53 students; 58%) and a life-like realistic simulation (*n* = 11/53 students; 21%). Many students also mentioned engagement and motivation (*n* = 29/53 students; 55%), the possibility of seeing different types of wounds in general (*n* = 19/53 students; 36%) and comparing them side-by-side (*n* = 9/53 students; 17%). An important advantage was also the increased practice of palpation and probing skills in deep wounds and wounds with cavities (*n* = 34/53 ­students; 64%) with the models (Table 2).

Our results suggest that 3D silicone wound models may improve the learning outcomes of the undergraduate teaching of chronic wounds by providing students with realistic, interactive and comprehensive simulation-based learning experiences. The 3D training sessions enabled new perspectives on the appearance of chronic wounds and the possibility of simultaneously seeing and palpating many types of wounds. They may also increase student motivation and function as practicable memory aids. However, our observations reflect student perceptions, not measured outcomes. In addition to the manual qualitative analysis of questionnaire data (e.g. key words), we decided to use the university’s own AI tool to achieve a more comprehensive and systematic analysis with an AI-based synthesis of the data. The use of AI in qualitative analyses is not new but, to our knowledge, has not been previously reported in studies of dermato­logy education.^4^

Research on the use of 3D models in dermatology education is very limited and has concentrated on suture training, recognition of skin conditions and patient education in surgery.^5,6^ Nevertheless, models have been traditional components of dermatology teaching and some studies have shown that they may improve lesion recognition and long-term learning outcomes.^7^ For example, lessons learned during the COVID-19 pandemic on the use of 3D models have been implemented permanently into teaching curricula so that students can benefit from the haptic experiences that such models provide.^8^

We found that 3D models had very promising potential in the diversification of the training of chronic wounds to include repeated tactile (e.g. palpation) and procedural (e.g. probing) tasks. To further improve their effectiveness, incorporating contextual information, expanding the variety of models and enhancing collaborative learning opportunities could be added. These improvements may lead to a more integrated and holistic approach to teachings of chronic wounds and better preparing students for clinical practice.^9^

The limitations of our study were the relatively small student cohort, relatively low participation rate with possible selection bias, the self-reported questionnaire and the missing data on objective learning outcomes. In addition, the parallel training design with dermatology courses makes it difficult to assess the independent effects of models. However, we could demonstrate a high practicability, student satisfaction and potential of integration into traditional teachings.^10^ Modern moulages, for example 3D silicone wound ­models, may have the potential to fill the pedagogical gap between contact teachings and digital education in dermatology and other fields of medicine.^11^ In any case, they seem to enhance student satisfaction and have positive effects on motivation to learn.

## Supplementary Material

vzag115_Supplementary_Data

## Data Availability

The complete data of the study are available in the Supporting Information.
