# Supplementary material for: Improving undergraduate teaching of chronic wounds with three-dimensional silicone models: a Finnish questionnaire-based cohort study
Source: Skin Health Dis. 2026 Jul 14;6(5):831–4. doi: 10.1093/skinhd/vzag115 (PMC13623429; doi:10.1093/skinhd/vzag115)
Supplement: Supplementary file 1 [file vzag115_supplementary_data.zip › Qualitative analysis_artificial intelligence.docx]

**Qualitative analyses (CurreChat®)**

**https://curre.helsinki.fi/chat, https://openai.com**

**Question no. 7**

From a teaching perspective, what were the advantages of using rubber models? How did they enhance your learning experience compared to other methods?

**Question no. 8**

What aspects of the rubber models could be improved to enhance the learning experience? How would you suggest making these improvements?

**Prompt:**

Pedagogical study: Integration of 3D wound models into traditional and online undergraduate teachings – a Finnish questionnaire-based cohort study

Analysis of students' answers to a questionnaire with open questions. Please make a qualitative analysis! 53 undergraduate students took part.

**Messages:**

Please make a qualitative analysis from a pedagogical perspective!

Question:

From a teaching perspective, what were the advantages of using rubber models? How did they enhance your learning experience compared to other methods?"

Answers:

Clinical reasoning, practice, clinical skills

Visuality, practice

Look real

Fun to examine ja measure

Without history one had to examine and interpret only the visual findings

3D, pictures do not always tell everything

Better perception of cavities, hypergranulation

Palpable, 3D

Good visualization

I think it's a really good addition to learning, although it always looks a bit different in real patients.

It is possible to systematically display wounds at different stages. For example, cavities and hypergranulation are 3D phenomena that are best seen in a model or in real life.

wound palpation, measurement, probing

Variation, probe measurement training

To illustrate the different wounds!

That you could touch them without any problems!

Also illustrate the wound in three dimensions!

3D visualization helps to visualize different types of changes. More senses are used for perception (vision, unto). You get to try probing and other skills before you get to the patient.

Concrete!

Concrete examples!

Visualization, memory imprint

It was good to see the different types of wounds side by side. Well suited for training in cavity and wound probing and palpation.

The models helped me to visualize things that I have never quite understood through pictures. It is useful to see many types of wounds in the models, because the patients we go to see bedside will occasionally have one type of wound and you don't get to see all the features of different types of wounds.

You can actually see what the wound looks like. To take your time to evaluate the wounds. Practice measuring of wounds.

I've never seen this type of wound as in the models.

You have to test for yourself how the wound feels. Palpation (hard, soft). Outlined how, for example, cavities could be explored. Helps to visualize the structure of tissue (e.g. fibrin).

An examination from the wound level was possible. Different viewing angles were also made possible. It was also useful to practice palpating the edges of the wound.

All types of wounds were clearly represented.

Compared to a 2D image, the models really add depth. The cavity model illustrates how to take measurements.

I saw plenty of different types of wounds quickly.

I got visual stimuli from different types of wounds. I saw so many different types of wounds that I did not have the possibility to see during bedside teaching in patients.

Informative, easy to illustrate wound types.

Able to compare different types of wounds side by side

Appearance, different types of wounds

Three-dimensional perception (cavities, hypergranulation), realistic appearance

Can compare the appearance and typical features of wounds, practice of wound cleaning with dermal curette

Interact with the models and the teacher.

You could feel how the wounds felt and see the wounds in 3D

**Question:**

What aspects of the rubber models could be improved to enhance the learning experience? How would you suggest making these improvements?"

**Answers:**

Patient history, background data, ulcer site

History, site

Adding context would improve learning, clinical practice, patterns, 3D relief

Only small part of patient, history is missing

Background, pulse status, history

More context information: site, environment, the general status of the patient

Include patient case, context

The interpretation could be improved if the ulcer site would be available

Would also work for skin tumors

Needs to be used more in teachings

Skin tumors would work as well, patient history needs to be included

The infected model could perhaps be even more clearly infected, so that I can more easily identify it in the future.

In lessons, for example, you could first think about questions with a partner.

More different models!

Help to refresh the issue. You can compare patient cases with rubber models.

There could be even clearer answers and a distinction between different types of wounds.

More models of different kind of wounds.

More models!

More models!

More explanations of wound findings (e.g. model wound) and the most common etiologies and examples of treatment options.

More models. Also of more models on different types of tumors.

It would have been useful if there had been even more different types of wound models.

More models of skin tumors.

More 3D models, so that all students can study them independently.

Could 3D models also be used to teach other topics?

More models of deep ulcers, e.g., contact with bones or tendons, to practice the probing of wounds

**Qualitative analysis (CurreChat) 13.04.2025, https://curre.helsinki.fi/chat, https://openai.com**

**Introduction**

The integration of 3D rubber wound models into undergraduate teaching has sparked interest in how such models can enhance clinical education. This qualitative analysis examines the responses of 53 Finnish undergraduate students regarding the pedagogical advantages and potential improvements of using these models. The analysis focuses on understanding the educational benefits and identifying areas for enhancement.

**Advantages of Using Rubber Models**

**Enhanced Visual and Tactile Learning**

A recurring theme in the students' responses is the significant visual and tactile benefit provided by rubber models. Many students noted that these models offer a realistic and three-dimensional representation of wounds, which is not achievable through traditional 2D images. This realism aids in better understanding complex structures such as cavities and hypergranulation, which are crucial for clinical skills development.

**Active Learning and Interactivity**

The models allow students to engage actively with the material, promoting hands-on learning. Participants emphasized the importance of being able to palpate, probe, and measure wounds, which fosters a deeper understanding of wound characteristics and enhances clinical reasoning. This interactivity helps bridge the gap between theoretical knowledge and practical application.

**Memory and Perception**

Visual stimuli from the models help create memory imprints, aiding long-term retention of information. Students appreciated being able to see a variety of wound types side by side, which is often limited in bedside teaching. This comparative approach supports the development of diagnostic skills and a comprehensive understanding of wound features.

**Comprehensive Representation**

The models provide a broad representation of different wound types, enabling students to visualize and understand wounds they may not encounter during clinical rotations. This exposure is vital for developing a well-rounded clinical perspective and preparing students for diverse patient scenarios.

**Areas for Improvement**

**Contextual Information**

A notable suggestion for improvement is the inclusion of patient history and contextual data. Students expressed a need for background information, such as ulcer site, pulse status, and general patient condition, to enhance interpretation and clinical decision-making. Integrating case studies or patient scenarios with the models could provide a more holistic learning experience.

**Variety and Expansion**

Students called for a greater variety of models, including more types of wounds and skin tumors. Increasing the diversity of models would allow students to explore a wider range of clinical conditions and refine their diagnostic skills. Additionally, the suggestion to expand the use of 3D models to other topics indicates a demand for broader application in medical education.

**Clarity and Detail**

Improving the clarity and detail of the models, particularly infected wounds, was a common theme. Students recommended clearer distinctions between different wound types and more explicit explanations of findings, aetiologies, and treatment options. Enhancing the detail in models would improve identification skills and provide a more accurate representation of clinical conditions.

**Collaborative Learning**

The integration of collaborative activities, such as partner discussions or group analysis, was suggested to enrich the learning experience. Collaborative learning encourages critical thinking and allows students to benefit from peer insights, fostering a deeper understanding of complex clinical scenarios.

**Conclusion**

The qualitative analysis reveals that 3D rubber wound models significantly enhance undergraduate clinical education by providing realistic, interactive, and comprehensive learning experiences. To further improve their effectiveness, incorporating contextual information, expanding the variety of models, and enhancing collaborative learning opportunities are recommended. These improvements could lead to a more integrated and holistic approach to medical education, better preparing students for clinical practice.

**Qualitative Analysis from a Pedagogical Perspective**

**Question 1: Advantages of Using Rubber Models**

The responses to the first question indicate several advantages of using rubber models in dermatology courses:

1. **Enhanced Clinical Skills and Reasoning**:
   - Students highlighted that rubber models aid in clinical reasoning and practice. The tactile and visual aspects of the models allow for a more hands-on approach to learning, which is crucial in medical education.
   - The ability to examine and measure the models provides a practical aspect that enhances the students' clinical skills.
2. **Visual and Tactile Learning**:
   - The visuality of the models was frequently mentioned. Students appreciated the realistic appearance of the models, which helps bridge the gap between theoretical knowledge and real-life application.
   - The 3D nature of the models allows for better perception of wound characteristics such as cavities and hypergranulation, which cannot be fully appreciated through pictures alone.
3. **Engagement and Interaction**:
   - The models make learning more interactive and fun, which can increase student engagement and motivation.
   - The palpable nature of the models provides an additional sensory dimension to learning, enhancing the overall educational experience.

**Question 2: Suggested Improvements**

The responses to the second question suggest several areas for improvement:

1. **Inclusion of Patient Context**:
   - Many students felt that the learning experience could be enhanced by including patient history and background data. This contextual information would allow students to practice interpreting visual findings in conjunction with clinical history, simulating real-life scenarios more closely.
   - Suggestions included adding details about the ulcer site, pulse status, and the general status of the patient.
2. **Expanded Usage**:
   - Some students mentioned that the rubber models should be used more frequently in teachings. Increased exposure to these models could further enhance learning outcomes.
3. **Application to Other Conditions**:
   - There was a suggestion to expand the use of rubber models to other dermatological conditions, such as skin tumors. This could broaden the scope of practical learning in dermatology courses.

**Question 3: Additional Comments and Suggestions**

The responses to the third question were brief but provided additional insights:

1. **Positive Feedback**:
   - One student expressed gratitude, indicating overall satisfaction with the use of rubber models.
   - Another student reiterated the potential for using rubber models for teaching about skin tumors, reinforcing the idea of expanding their application.

**Question 4: Comments on Specific Questions**

The responses to the fourth question provided further elaboration on key points:

1. **Importance of Context**:
   - Students emphasized that adding context would significantly improve the learning experience. Understanding the clinical patterns and practicing clinical practice in a more holistic manner is crucial for developing comprehensive clinical skills.
   - The lack of patient history was noted as a limitation, suggesting that integrating this information would provide a more complete learning experience.

**Summary**

From a pedagogical perspective, the integration of rubber models into traditional dermatology teachings offers several advantages. These include enhanced clinical skills, improved visual and tactile learning, and increased student engagement. However, there is room for improvement, particularly in providing patient context and expanding the use of models to other dermatological conditions. Overall, students appreciate the use of rubber models and see potential for further enhancement of their learning experience through thoughtful integration and expansion.
