# Supplementary material for: Improving undergraduate teaching of chronic wounds with three-dimensional silicone models: a Finnish questionnaire-based cohort study
Source: Skin Health Dis. 2026 Jul 14;6(5):831–4. doi: 10.1093/skinhd/vzag115 (PMC13623429; doi:10.1093/skinhd/vzag115)
Supplement: Supplementary file 1 [file vzag115_supplementary_data.zip › Qualitative analysis_manual.docx]

**Qualitative analysis by hand (counting and ranking of key words of open questions answered by the students)**

**Question no. 7**

From a teaching perspective, what were the advantages of using rubber models? How did they enhance your learning experience compared to other methods?

Visual skills (visualization): 31 (58.5%)

Palpation and probing skills: 34 (64.2%)

Clinical skills, 3D perspective: 26 (49.1%)

Fun and motivation: 29 (54.7%)

Possibility to see different types of wounds: 19 (35.9%)

Practice (repetitions): 15 (28.3%)

Real-life imitation: 11 (20.8%)

Seeing different types of wounds side-by-side: 9 (17.0%)

**Question no. 8**

What aspects of the rubber models could be improved to enhance the learning experience? How would you suggest making these improvements?

Needs to be used more in teachings, more models: 39 (73.6%)

Including background information (patient history, wound site): 31 (58.5%)

Models also from skin tumors: 26 (49.1%)
